# Change in Viability and Function of Pancreatic Islets after Coculture with Mesenchymal Stromal Cells: A Systemic Review and Meta-Analysis

**DOI:** 10.1155/2020/5860417

**Published:** 2020-03-24

**Authors:** Xiaohang Li, Hongxin Lang, Baifeng Li, Chengshuo Zhang, Ning Sun, Jianzhen Lin, Jialin Zhang

**Affiliations:** ^1^Department of Hepatobiliary Surgery and Organ Transplant, First Affiliated Hospital, China Medical University, No. 155, Nanjing North Street, Shenyang, 110001 Liaoning Province, China; ^2^Department of Stem Cells and Regenerative Medicine, Shenyang Key Laboratory for Stem Cells and Regenerative Medicine, Key Laboratory of Cell Biology, Ministry of Public Health, and Key Laboratory of Medical Cell Biology, Ministry of Education, China Medical University, No. 77 Puhe Street, Shenbei New District, Shenyang, 110122 Liaoning Province, China

## Abstract

**Background:**

There is no clear consensus on the effect of coculture of islets with mesenchymal stem cells (MSCs) on islet function and viability.

**Methods:**

We conducted a meta-analysis of relevant studies to evaluate the effect of coculture of islets with MSCs on the function and viability of islets, both *in vitro* and *in vivo*. We searched PubMed, Embase, and Web of Science databases for all relevant studies that compared the effect of coculture of islets with MSCs on the function and viability of islets (language of publication: English; reference period: January 2000–May 2019). Data pertaining to islet function and viability, concentrations of some cytokines, and *in vivo* experimental outcomes were extracted and compared.

**Results:**

Twenty-four articles were included in the meta-analysis. In comparison to islets cultured alone, coculture of islets with MSCs was associated with a significantly higher islet viability [weighted mean difference (WMD), -15.59; -22.34 to -8.83; *P* < 0.00001], insulin level (WMD, -5.74; -9.29 to -2.19; *P* = 0.002), insulin secretion index (WMD, -2.45; -3.70 to -1.21; *P* = 0.0001), and higher concentrations of interleukin-6 (WMD, -1225.66; -2044.47 to -406.86; *P* = 0.003) and vascular endothelial growth factor (WMD, -1.19; -2.25 to -0.14; *P* = 0.03). Direct coculture of islets and MSCs significantly increased islet viability (WMD, -19.82; -26.56 to -13.07; *P* < 0.00001). In the *in vivo* experiments, coculture of islets with MSCs induced lower fasting blood glucose level (on postoperative days 21 and 28, WMD, 102.60; 27.14 to 178.05; *P* = 0.008 and WMD, 121.19; 49.56 to 192.82; *P* = 0.0009) and better glucose tolerance (blood glucose at 30 minutes after intraperitoneal injection of glucose, WMD, 85.92; 5.33 to 166.51; *P* = 0.04).

**Conclusion:**

Coculture of islets with MSCs improves insulin secretory function of islets and enhances islet viability. Direct coculture of two cells significantly increased islet viability. MSC-based strategy may be beneficial for clinical islet transplantation for type 1 diabetes in the future.

## 1. Introduction

The prevalence of diabetes mellitus (DM) is rapidly rising worldwide. Globally, an estimated 382 million people are suffering from DM, and the number is projected to reach 592 million by 2035 [1]. Type 1 diabetes mellitus (T1DM) is caused by autoimmune-mediated injury of the pancreatic *β* cells, which results in absolute insulin deficiency [2]. T1DM accounts for approximately 10% of all patients with DM. Since the adoption of the Edmonton protocol in 1999 [3], islet transplantation has become a promising treatment modality for T1DM patients with frequent hypoglycemic unawareness. Nevertheless, long-term graft survival after islet transplantation is still not satisfactory. The poor survival may be attributable to the instant blood-mediated inflammatory reaction (IBMIR), immunological rejection, and/or nonspecific inflammatory reaction. Human islets are usually transplanted into the liver through the portal vein under radiological guidance, which leads to their engraftment in the liver. The intrahepatic portal venous system is a hypoxic environment which may trigger innate immune responses [4], and this hypoxic condition may be further aggravated by embolization of the peripheral portal vein. These factors are believed responsible for the early and high loss of human islets after transplantation [5]. Recognition of these problems has prompted efforts to improve the engraftment survival rate after islet allotransplantation. Considerable effort has been invested to improve the outcomes; these include minimizing the loss of islet function during isolation and purification and search for a new site for transplantation instead of the portal vein [6]. Further optimization of this transplant technique is required prior to its clinical application.

Another research area to improve graft survival focuses on coculture of islets with mesenchymal stem cells (MSCs). MSCs are the most well-characterized adult stem cells. MSCs have evoked considerable research interest owing to their capacity for multipotent self-renewal and multilineage differentiation. MSCs have potent immunoregulatory properties [7] and thus represent an attractive therapeutic method in the context of islet transplantation. Cotransplantation of islets with MSCs is one of the strategies to improve islet viability and function [8, 9]. MSCs exist in the stromal fraction of numerous tissues (such as adipose, bone marrow, amniotic membrane, and umbilical cord) in multiple species [10]. In addition, MSCs have been shown to enhance viability and function of islets during coculture [11]. However, there are some opposite viewpoints [12]. In addition, there is no clear consensus as to whether the direct contact between MSCs and islets during coculture is essential to improve the viability and function of islets [13–16]. Therefore, we performed this meta-analysis to assess whether coculture of islets with MSCs improves the viability and function of islets and whether direct contact between MSCs and islets enhances the function of islets.

A similar meta-analysis by de Souza et al. was published in 2017 [17]; however, the authors did not include in vivo experimental outcomes. Our meta-analysis includes five additional studies [15, 18–21], and we compare a wider range of outcomes including those of in vivo experiments. We also draw some conclusions which are different from the previous one. Therefore, we believe that our study provides novel insights into the effect of coculture of islets with MSCs on the function and viability of islets.

## 2. Methods

This review was conducted in accordance with the PRISMA statement [22].

### 2.1. Literature Search

The electronic databases (PubMed, Embase, and Web of Science) were searched for relevant literature published between January 2000 and May 2019 in English language. The following key words/medical subject headings (MeSH) were used for the search: (“mesenchymal stem/stromal cell” OR “mesenchymal cell” OR “mesenchymal stem cell transplantation” OR “mesenchymal cell research”) AND (“pancreas/pancreatic islet” OR “islets of Langerhans” OR “pancreas/pancreatic islet transplantation” OR “islet transplantation”). The “related articles” function was used to widen the search, and the references of the retrieved articles were manually screened to identify potential eligible literature.

### 2.2. Assessment of Study Eligibility

Two investigators independently evaluated all the articles by reviewing the titles and abstracts; if necessary, the full text of the articles was reviewed. Discrepancies, if any, were resolved by discussion or in consultation with a senior reviewer. Eligible studies were selected according to the following inclusion criteria: (1) original research articles that compared the outcomes of pancreatic islets cultured alone with those of islets and MSCs coculture and (2) studies that reported at least one of the outcomes of interest (see below) and the mean (±standard deviation) values for continuous variables of interest. The exclusion criteria were literature reviews and case reports, studies published in languages other than English, and duplicate publications.

The outcomes of interest included islet viability and function in vitro, the concentrations of cytokines, and the in vivo experimental results. If the article did not contain complete data, the authors were contacted to obtain complete data using a detailed data extraction form. For the direct coculture model, islets were seeded directly onto the MSC monolayer in a plate. For the indirect coculture model, cell culture transwells with a semipermeable membrane to which islets were added were inserted into each well with the MSC monolayer. The meta-analysis is based on the studies published previously; therefore, ethical approval was not required.

### 2.3. Data Extraction and Quality Assessment

Two investigators independently extracted the following data from each included study: first author, country, year of publication, islet origin (human/murine/pig), MSC origin (human/murine), type of MSCs (bone marrow/umbilical cord blood/adipose tissue/kidney-derived MSCs), method of coculture (indirect/direct/coencapsulated), culture duration, results of islet viability and function (as mean ± SD, or estimated by graphics presented in the articles), and the concentrations of cytokines secreted by islets (mean ± SD). For studies that included in vivo experiments, the following data were also extracted: species of donor and recipient, number of transplanted islets and MSCs, site of transplantation, and the outcomes posttransplantation, which included the level of fasting blood glucose (FBG) and results of intraperitoneal glucose tolerance test (IPGTT).

For the viability outcomes, we classified the staining dyes based on whether they stained viable or dead cells, e.g., fluorescein diacetate (FDA)/propidium iodide (PI), acridine orange (AO)/PI, Syto-Green/ethidium bromide (EB), ethidium homodimer-1 (EH-1)/calcein AM, or trypan blue. For the islet function outcomes, the analyzed data included insulin secretion index (ISI; rate of high- to low-glucose-stimulated insulin secretion) and the level of insulin secreted after glucose stimulation when the basic level of insulin was not provided. For the posttransplantation outcomes, we compared the levels of FBG at different days posttransplantation. The outcomes of IPGTT were also compared.

Grading of Recommendation Assessment, Development and Evaluation (GRADE) guidelines [23] were used to evaluate the overall quality of the included studies. According to GRADE recommendations, the quality of evidence was categorized into 4 types: high, moderate, low, or very low, based on the study design, imprecision, inconsistency, and indirectness.

### 2.4. Statistical Analysis

The meta-analysis was performed using the Cochrane Collaboration Review Manager 5.3. For continuous variables, statistical analysis was carried out using the weighted mean differences (WMD) with 95% confidence intervals (CI) as the summary statistic. Results were considered statistically significant at *P* < 0.05, if the 95% CI did not include the value zero. First, homogeneity among the included studies was assessed using the *Q*-test statistic. In case of significant heterogeneity, a random effects model was used for meta-analysis; a fixed effects model was used in case of lack of significant heterogeneity [24].

Due to considerable variability among the included studies with respect to experimental conditions and parameters, significant heterogeneity was expected. To further control for heterogeneity, subgroup meta-analyses were performed to assess the possible association between different variables (such as coculture methods) and outcomes.

## 3. Results

### 3.1. Literature Search

**Figure 1:**
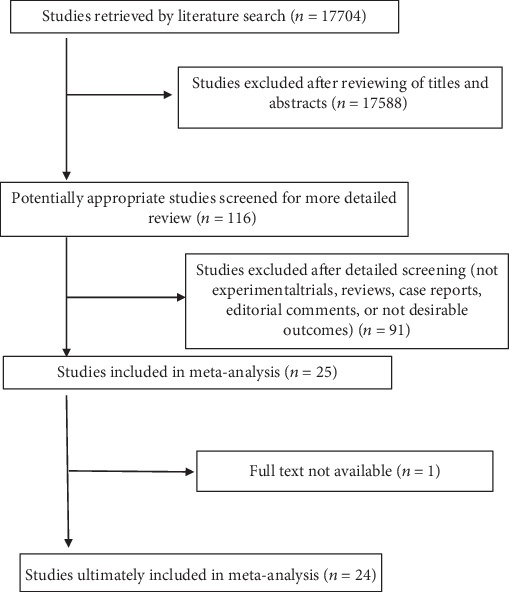
Schematic illustration of the literature search and study selection criteria.

A total of 17704 articles were retrieved on database search. After a meticulous review of titles and abstracts, 17588 studies were excluded. After further detailed review, another 91 studies were excluded due to ineligible study design, lack of outcomes of interest, or experimental trials. Twenty-five studies qualified the inclusion criteria [9, 11–16, 18–21, 25–38]. However, one article was excluded due to lack of availability of full text [32]; therefore, 24 studies were included in the meta-analysis (Figure 1). The characteristics of the included studies are presented in Table 1. Since some studies analyzed different periods of culture, culture methods, MSC tissue origins, and concentrations of MSCs, some studies were included more than once in the analysis.

### 3.2. Quality Assessment of the Included Studies

The quality of the studies included in the meta-analysis was assessed using the GRADE [23]. Since the studies included in the meta-analysis were not blinded, the evidence was classified as having low to very low quality according to the GRADE recommendations,

### 3.3. Assessment of Islet Function In Vitro

**Figure 2:**
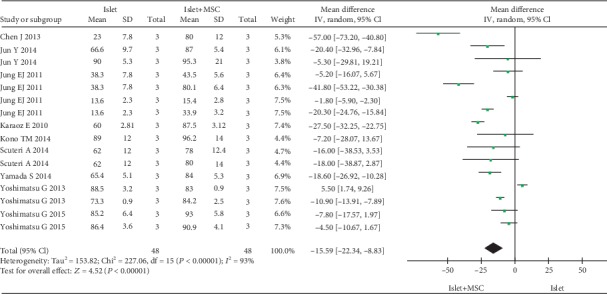
Forest plot of meta-analysis of islet viability, comparing islets cultured alone with islets cocultured with MSCs.

**Figure 3:**
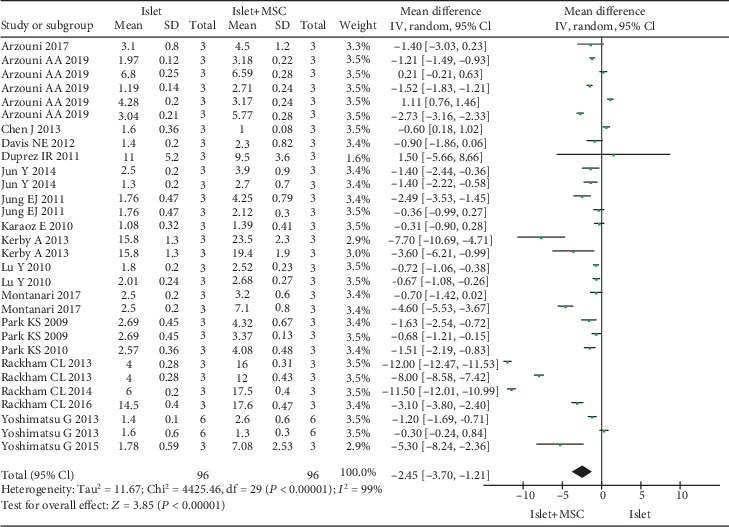
Forest plot of meta-analysis of islet insulin secretion index, comparing islets cultured alone with islets cocultured with MSCs.

**Figure 4:**
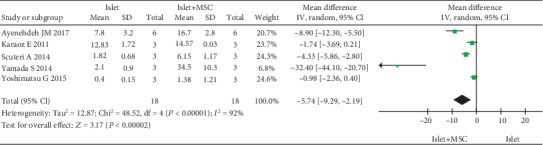
Forest plot of meta-analysis of islet-secreted insulin level, comparing islets cultured alone with islets cocultured with MSCs.

Three parameters were used to assess the function of islets in vitro: viability, ISI, and the level of insulin secreted after glucose stimulation. A total of 10 studies reported data pertaining to islet viability; however, concrete data pertaining to islet viability was not available for one study. Therefore, nine studies were included in the quantitative pooled analysis for viability of islets. Seventeen studies reported ISI; four studies reported the level of insulin secretion. In comparison to islet cultured alone, islet cocultured with MSCs showed significantly increased islet viability (WMD, -15.59; -22.34 to -8.83; *P* < 0.00001) (Figure 2), ISI (WMD, -2.45; -3.70 to -1.21; *P* = 0.0001) (Figure 3), and insulin secretion (WMD, -5.74; -9.29 to -2.19; *P* = 0.002) (Figure 4). However, subgroup analysis disaggregated by a coculture method yielded a different result pertaining to islet viability. Indirect coculture with MSCs was not associated with significantly increased islet viability (WMD, -1.14; -7.82 to 5.54; *P* < 0.74), while direct coculture with MSCs was associated with significantly increased islet viability (WMD, -19.82; -26.56 to -13.07; *P* < 0.00001) (Table 2). However, on subgroup analysis, both direct and indirect cocultures with MSCs were associated with significantly improved ISI (WMD, -2.42; -3.94 to -0.89; *P* = 0.002; WMD, -2.54; -4.79 to -0.28; *P* = 0.03) (Table 2).

### 3.4. Concentration of Cytokines

Five studies had compared the concentration of cytokines in the supernatant of the culture medium [four studies for vascular endothelial growth factor (VEGF), three studies for interleukin-6 (IL-6), and three studies for tumor necrosis factor-*α* (TNF-*α*)]. The concentrations of VEGF and IL-6 in the supernatant of islets cocultured with MSCs were significantly higher than those in islet cultured alone (WMD, -1.19; -2.25 to -0.14; *P* = 0.03 and WMD, -1225.66; -2044.47 to -406.86; *P* = 0.003); however, the concentration of TNF-*α* in the supernatant of islet cultured alone was higher although the difference was not statistically significant (WMD, 2.70; -0.50 to 5.91; *P* = 0.10). Moreover, subgroup analysis after exclusion of indirect coculture showed similar results (Table 3).

### 3.5. Assessment of Function of Islets In Vivo

Eight studies that reported the level of FBG and IPGTT after transplantation were included in the meta-analysis (Table 4). The reported variables include the number of transplanted cells, transplantation site, and origin of donor and recipient. A total of six studies reported the level of FBG on postoperative days 7, 14, 21, and 28, while five studies had reported the results of IPGTT. Blood glucose levels were measured at 30, 60, 90, and 120 minutes after intraperitoneal injection of glucose solution (2 G 20% glucose solution per kg body weight). The level of FBG on postoperative days 21 and 28 in islet alone transplantation group was significantly greater than that in the islet and MSC cotransplantation group (Table 5). On subgroup analysis, the level of FBG on postoperative days 7, 14, and 28 in the islet alone transplantation group was significantly greater than that in the cotransplantation group wherein islets and MSCs were unencapsulated together prior to transplantation (Table 5). Blood glucose levels at 30 and 60 minutes after intraperitoneal injection of glucose in the islet alone transplantation group were significantly higher than those in the islet and MSC cotransplantation group. Subgroup analysis based on whether islet and MSCs were encapsulated together before transplantation showed a similar result (Table 6).

## 4. Discussion

In addition to the quantity, the function and vitality of islets are also key determinants of the success of islet transplantation. The remarkable advances in islet isolation and purification techniques have augmented islet yield, improved islet function, and produced better outcomes after single donor islet allotransplantation by ensuring an increased functional *β* cell mass [39–42]. However, the long-term outcomes of islet transplantation are largely disappointing. In the recent era, the reported insulin independence at 3 years postislet transplantation was only 44% [43]. This may be attributable to immunological rejection and drug toxicity, despite the availability of improved immunosuppressive regimens. Therefore, ongoing research to improve the long-term outcomes of islet transplantation by inhibiting and modulating alloimmune and autoimmune processes is a key imperative.

MSCs have previously been shown to help preserve *β* cell function in type 1 diabetes [44]; in addition, MSCs have the ability to modulate both innate and adaptive immune responses [7]. Therefore, several studies have investigated the outcomes of coculture of MSCs with islets. However, there is no clear consensus on the effect of MSCs in improving outcomes of islet transplantation and the optimal coculture model for this purpose. The results of our meta-analysis suggest that coculture of islets with MSCs improves the insulin secretory function of islets and enhances islet viability when compared with islets cultured alone. Several studies have shown that MSCs have the ability to enhance insulin secretion [45, 46], although the underlying mechanism is not fully elucidated. Rackham et al. demonstrated that mouse adipose and kidney-derived MSCs produce annexin A1 (ANXA1), also known as lipocortin 1, with well-documented anti-inflammatory properties [47], which exhibited a beneficial effect on mouse islet insulin secretion [35]. In addition, MSCs may improve islet survival by modulating the levels of signal molecules. Besides HSP-32, which is known to have a cytoprotective effect on islets, some antiapoptotic signaling molecules, such as XIAP [48], Bcl-2 [49], and Bcl-xL, were shown to have greater expression in islets cocultured with human cord blood-derived MSC than in islets alone [26]. In addition, MSCs secrete many kinds of cytokines via both paracrine and autocrine mechanisms, which may help improve islet viability [50, 51].

Our study showed that the concentrations of VEGF and IL-6 in the supernatant of islets cocultured with the MSC group were significantly higher than those in the islet cultured alone group; however, the concentration of TNF-*α* in the supernatant of the islet cultured alone group was higher. Park et al. considered that VEGF signaling plays a key role in mediating the protective effect of MSCs [26]. Moreover, VEGF suppresses the apoptosis of granulosa cells by inhibiting the release of caspase-activated Dnase [52]. VEGF is also known to promote vasculogenesis and angiogenesis. IL-6, another cytokine secreted by MSCs, is believed to protect pancreatic *β* cells from apoptosis by direct stimulation of autophagy [53, 54]. Therefore, the improved viability and function of islets cocultured with MSCs may be partly attributed to the high concentrations of VEGF and IL-6. Nevertheless, TNF-*α* as a proinflammatory cytokine which is cytotoxic to *β* cells was shown to exhibit a disruptive effect on insulin biosynthesis [55]. The lower concentration of TNF-*α* in the supernatant of islets cultured with MSCs indirectly indicates a protective effect of MSCs on islets. Although most studies suggest that the cytoprotective cytokines secreted by MSCs have a beneficial effect on islet, there is no consensus as to which kind of islet-MSC coculture configurations has a greater beneficial effect on the islets. In the study by Rackham et al., direct islet-MSC coculture configuration improved islet function [14], which was likely attributable to the increased concentration of MSC-secreted cytokines as compared to that observed with the use of indirect coculture. Similarly, Jung et al. and Luo et al. proposed that physical contact between islets and MSCs may help preserve the structural integrity of islets [13, 56], which improves the function and viability of islets. According to another school of thought, the soluble cytokines secreted by MSCs rather than physical contact may play a more important role in this respect. Our findings suggest that direct coculture with MSCs, rather than indirect coculture, significantly increased islet viability; however, both coculture configurations had a comparable effect in improving islet function. This conclusion is not consistent with that of meta-analysis by de Souza et al. In their study, the improvement in viability associated with indirect coculture of islets with MSCs was significantly greater than that observed with direct coculture. Since our meta-analysis included more studies, we believe that direct coculture, which allows for physical contact between the two cell types, may present more superior effect.

We compared the levels of FBG and results of IPGTT after islet/MSC transplantation in order to evaluate the function of islet in vivo. We found that the level of FBG on postoperative days 21 and 28 was higher in the islet alone transplantation group. Interestingly, subgroup analysis showed that the level of FBG on postoperative days 7, 14, and 28 was significantly lower when unencapsulated islets and MSCs were cotransplanted. Due to inflammation, hypoxic ischemic environment, and immunological factors, early loss of functional *β* cells is common in the first hours/days after islet transplantation [57]. MSCs may play a part in immunological modulation and angiogenesis upon cotransplantation with islets. As mentioned above, VEGF secreted by MSCs can promote angiogenesis, which is particularly beneficial for improving the function and viability of the transplanted islets. MSCs can also suppress immunological rejection by inhibiting cytotoxic T-cell (Tc) activity [58] and reducing lymphocyte infiltration at the graft site [59]. In addition, the MSCs provide abundant physical and nutritional support for the transplanted islets which were extracted from their dense vasculature and extracellular matrix; consequently, the improved microenvironment may be more suitable for the survival of islets. A new therapeutic strategy that entails encapsulating islets with biomaterials to overcome the immunological and inflammatory attack has been reported [26]. However, in our study, the use of encapsulated islets and MSCs was not associated with lower FBG as compared to that achieved with unencapsulated islets. We believe that this may be related to the heterogeneity among the included studies with respect to encapsulation biomaterials (hydrogel, alginate, or polyvinyl alcohol) and the number of transplanted MSCs and islets. Furthermore, IPGTT demonstrated more severe impairment of glucose tolerance in the islet alone transplantation group; in addition, subgroup analysis based on whether islet and MSCs were encapsulated together showed a similar result. Thus, on comparing the in vivo experimental outcomes, we found that MSCs improve the function of islets upon cotransplantation.

Some limitations of our study should be considered while interpreting our findings. First, since studies included in our meta-analysis were not blinded, the quality of evidence was low. The strength of proof is relatively low, and due caution should be exercised while drawing conclusions. Second, there was considerable variability between the included studies with respect to the origin and types of MSCs, the origins of islets, and the duration of coculture. Also, the included studies involved different kinds of transplantations (syngeneic, allogeneic, or xenotransplantation) and transplantation sites. All these factors contributed to significant heterogeneity. In addition, different coculture methods were used in the included studies; however, we performed subgroup analysis to minimize the influence of heterogeneity. Third, the methods used for the assessment of islet viability and function were not completely consistent across studies; in addition, the assessments were performed at different time points. Despite the limitations mentioned above, we believe that our study obviously decreases the heterogeneity by subgroup analysis and provides important information regarding change in viability and function of pancreatic islets after coculture with MSCs.

## 5. Conclusions

Our study demonstrated that MSCs can significantly improve the viability and function of islets when they are directly or indirectly cocultured. MSCs may exert their protective effect by modulating the secretion of cytokines, such as VEGF, TNF-*α*, and IL-6. Direct coculture of islets and MSCs augments islet viability; this phenomenon may be attributed to the physical contact between the two cell types which helps preserve the structural integrity of islets. Moreover, cotransplantation of MSCs can significantly improve islet function in vivo, which may be ascribed to the effect of MSCs on immunological modulation and angiogenesis. Therefore, a MSC-based strategy might represent a major step forward towards clinical islet transplantation for type 1 diabetes.

## Figures and Tables

**Table 1:** Characteristics of the eligible studies included in the meta-analysis.

Author [reference]	Year	Outcome evaluated	Technique used	Viability test	Islet origin/MSC tissue origin	Type of coculture	Time of coculture
Arzouni et al. [18]	2017	ISI	Radioimmunoassay	—	Human/hAD-MSC	Direct	4 d
Arzouni et al. [19]	2019	ISI	Radioimmunoassay	—	Human, mouse/hAD-MSC, hBM-MSC, mAD-MSC, mKidney-MSC	Direct	1 d/2 d/3 d
Chen et al. [38]	2013	Viability, ISI	Radioimmunoassay	AO/PI	Rat/rBM-MSC	Direct	2 w
Davis et al. [27]	2012	ISI	ELISA	—	Mouse/mBM-MSC	Encapsulation	7 d
Duprez et al. [12]	2011	ISI	ELISA	—	Human/hBM-MSC	Direct	2 d
Jun et al. [28]	2014	ISI, viability	ELISA	Calcein AM/ethidium homodimer-1	Rat/rAD-MSC	Encapsulation	7 d/14 d
Jung et al. [13]	2011	Viability, VEGF, TNF-*α*, ISI	ELISA	FDA/PI	Rat/rBM-MSC	Indirect/direct	1 w/4 w
Karaoz et al. [29]	2010	Viability, IL-6, ISI	ELISA	FDA/PI	Rat/rBM-MSC	Indirect	2 w
Karaoz et al. [11]	2011	Insulin	ELISA		Rat/rBM-MSC	Direct	16 d
Kerby et al. [30]	2013	ISI	Radioimmunoassay		Mouse/mKidney-MSC	Encapsulation	3 d
Kono et al. [31]	2014	Viability, VEGF	—	EH 1/calcein AM	Mouse/hAD-MSC	Indirect	6 d
Lu et al. [33]	2010	ISI	ELISA	—	Rat/rBM-MSC	Direct	3 d/4 d
Ayenehdeh et al. [20]	2017	Insulin	ELISA	—	Mouse/mAD-MSC	Encapsulation	—
Montanari et al. [15]	2017	ISI	ELISA	—	Human/hBM-MSC	Indirect/direct	3 d
Park et al. [25]	2009	ISI, VEGF, IL-6, TNF-*α*	ELISA	—	Human/hBM-MSC, hUCB-MSC	Indirect	2 d
Park et al. [26]	2010	ISI, viability	ELISA	AO/PI	Mouse/hUCB-MSC	Indirect	2 d
Rackham et al. [14]	2013	ISI	Radioimmunoassay	—	Mouse/mKidney-MSC	Indirect/direct	3 d
Rackham et al. [34]	2014	ISI	Radioimmunoassay	—	Mouse/mAD-MSC	Direct	3 d
Rackham et al. [35]	2016	ISI	Radioimmunoassay		Mouse/mAD-MSC	Direct	3 d
Scuteri et al. [16]	2014	Viability, insulin	ELISA	Calcein AM	Rat/rBM-MSC	Indirect/direct	1 w
Yamada et al. [36]	2014	Viability, IL-6, VEGF, TNF-*α*, insulin	ELISA	Trypan blue staining	Pig/hAD-MSC	Indirect	2 d
Yoshimatsu et al. [37]	2013	Viability, ISI	ELISA	Syto-Green/EB	Rat/rBM-MSC	Direct/encapsulation	3 d
Yoshimatsu et al. [9]	2015	Viability, ISI, insulin	ELISA	Syto-Green/EB	Mouse/mBM-MSC	Direct	1 d/4 d
Ren et al. [21]	2019	—	—	—	Mouse/mAD-MSC	Cotransplantation	—

Note. MSC = mesenchymal stromal cells; ISI = insulin stimulation index; FDA/PI = fluorescein diacetate/propidium iodide; EB = ethidium bromide; EH 1 = ethidium homodimer-1; VEGF = vascular endothelial growth factor; IL-6 = interleukin-6; TNF-*α* = tumor necrosis factor-*α*; h = human; m = mouse; BM = bone marrow; UCB = umbilical cord blood; AD = adipose; d = day; w = week.

**Table 2:** Meta-analysis of islet viability and ISI in subgroups.

Outcome	Subgroup	Studies	Meta-analysis		*P*	*I* ^2^ (%)
			Mean difference	95% CI		
Viability	Direct coculture	12	-19.82	-26.56~-13.07	<0.00001	89
	Indirect coculture	4	-1.14	-7.82~5.54	0.74	74
ISI	Direct coculture	21	-2.42	-3.94~-0.89	0.002	99
	Indirect coculture	9	-2.54	-4.79~-0.28	0.03	99

Note. ISI = insulin stimulation index; CI = confidence interval.

**Table 3:** Meta-analysis of cytokines outcomes in studies that compared islets cultured alone with islets cocultured with MSCs.

Outcome	Studies reported outcome		Meta-analysis		*P*	*I* ^2^ (%)
	Number	Unit	Mean difference	95% CI		
VEGF	4	pg/*μ*L	-1.19	-2.25~-0.14	0.03	100
Excluding indirect	4	pg/*μ*L	-1.82	-3.34~-0.30	0.02	100
TNF-*α*	3	pg/mL	2.70	-0.50~5.91	0.10	91
Excluding indirect	3	pg/mL	1.86	-3.53~7.24	0.50	93
IL-6	3	pg/mL	-1225.66	-2044.47~-406.86	0.003	100

Note. CI = confidence interval; VEGF = vascular endothelial growth factor; IL-6 = interleukin-6; TNF-*α* = tumor necrosis factor-*α*.

**Table 4:** Characteristics of studies included in the meta-analysis that performed islet transplantation *in vivo*.

Author [reference]	Year	Outcome evaluated	Number of transplantation cell		Encapsulation	Transplantation site	Islet origin	Recipient origin
			Islet	MSCs				
Davis et al. [27]	2012	IPGTT	600 IEQ	600	Yes	Peritoneal cavity	SD rat	Balb/C mouse
Karaoz et al. [29]	2011	FBG	350 IEQ	450-500	Yes	Peritoneal cavity	C57BL/6J	C57BL/6J
Ayenehdeh et al. [20]	2017	FBG	100 IEQ	2 × 10^5^	No	Peritoneal cavity	Balb/C	C57BL/6
Montanari et al. [15]	2017	IPGTT	4500–5000 IEQ	450-500	Yes	Peritoneal cavity	Human	C57BL/6 mouse
Rackham et al. [14]	2013	FBG, IPGTT	100 IEQ	2 × 10^5^	No	Underneath the kidney capsule	C567BL/6	C567BL/6
Ren et al. [21]	2019	FBG	150–225IEQ	1 × 10^6^	No	Underneath the kidney capsule	C57BL/6	C57BL/6
Yamada et al. [36]	2014	FBG, IPGTT	1500 IEQ	1 × 10^6^	Yes	Peritoneal cavity	Wistar rat	Balb/C mouse
Yoshimatsu et al. [9]	2015	FBG, IPGTT	500 IEQ	5 × 10^5^	No	Femur muscle	Balb/C	Balb/C

Note. FBG = fasting blood glucose; IPGTT = intraperitoneal glucose tolerance test; IEQ = islet equivalent.

**Table 5:** Meta-analysis of fasting blood glucose level in studies that performed islet transplantation *in vivo*.

Outcome	Meta-analysis		*P*	*I* ^2^ (%)
	Mean difference	95% CI		
FBG-POD7	75.97	-10.79~162.74	0.09	99
Encapsulation	5.05	-153.86~63.96	0.95	98
Unencapsulation	127.47	47.17~207.77	0.002	99
FBG-POD14	97.65	-1.82~197.12	0.05	99
Encapsulation	20.15	-145.74~186.05	0.81	98
Unencapsulation	154.69	46.13~263.25	0.005	99
FBG-POD21	102.60	27.14~178.05	0.008	99
Encapsulation	117.19	-44.39~278.76	0.16	98
Unencapsulation	93.20	-25.59~212.00	0.12	99
FBG-POD28	121.19	49.56~192.82	0.0009	99
Encapsulation	105.73	-66.80~278.25	0.23	99
Unencapsulation	133.85	29.47~238.23	0.01	99

Note. FBG = fasting blood glucose; POD = postoperative day; CI = confidence interval.

**Table 6:** Meta-analysis of IPGTT in studies performing islet transplantation in vivo.

Outcome	Meta-analysis		*P*	*I* ^2^ (%)
	Mean difference	95% CI		
BG-30 min	85.92	5.33~166.51	0.04	99
Encapsulation	132.60	-1.06~266.27	0.05	99
Unencapsulation	10.07	-16.26~36.40	0.45	45
BG-60 min	100.47	37.39-163.55	0.002	98
Encapsulation	123.21	-14.56~260.99	0.08	99
Unencapsulation	71.26	27.83~114.70	0.001	81
BG-90 min	57.59	-44.23~159.40	0.27	99
Encapsulation	123.41	-8.75~255.56	0.07	99
Unencapsulation	-41	-114.45~32.45	0.27	73
BG-120 min	66.77	-48.25~181.79	0.26	99
Encapsulation	139.47	11.81~267.13	0.03	99
Unencapsulation	-42.35	-108.67~23.98	0.21	99

Note. CI = confidence interval; BG = blood glucose.

## References

[1] Guariguata L., Whiting D. R., Hambleton I., Beagley J., Linnenkamp U., Shaw J. E. (2014). Global estimates of diabetes prevalence for 2013 and projections for 2035. *Diabetes Research and Clinical Practice*.

[2] Aguayo-Mazzucato C., Bonner-Weir S. (2010). Stem cell therapy for type 1 diabetes mellitus. *Nature Reviews Endocrinology*.

[3] Shapiro A. M., Lakey J. R., Ryan E. A. (2000). Islet transplantation in seven patients with type 1 diabetes mellitus using a glucocorticoid-free immunosuppressive regimen. *The New England Journal of Medicine*.

[4] van der Windt D. J., Bottino R., Casu A., Campanile N., Cooper D. K. (2007). Rapid loss of intraportally transplanted islets: an overview of pathophysiology and preventive strategies. *Xenotransplantation*.

[5] Hardstedt M., Lindblom S., Karlsson-Parra A., Nilsson B., Korsgren O. (2016). Characterization of innate immunity in an extended whole blood model of human islet allotransplantation. *Cell Transplant*.

[6] Pepper A. R., Bruni A., Pawlick R. L. (2016). Long-term function and optimization of mouse and human islet transplantation in the subcutaneous device-less site. *Islets*.

[7] Ghannam S., Bouffi C., Djouad F., Jorgensen C., Noel D. (2010). Immunosuppression by mesenchymal stem cells: mechanisms and clinical applications. *Stem Cell Research & Therapy*.

[8] Borg D. J., Weigelt M., Wilhelm C. (2014). Mesenchymal stromal cells improve transplanted islet survival and islet function in a syngeneic mouse model. *Diabetologia*.

[9] Yoshimatsu G., Sakata N., Tsuchiya H. (2015). The co-transplantation of bone marrow derived mesenchymal stem cells reduced inflammation in intramuscular islet transplantation. *PLoS One*.

[10] da Silva Meirelles L., Chagastelles P. C., Nardi N. B. (2006). Mesenchymal stem cells reside in virtually all post-natal organs and tissues. *Journal of Cell Science*.

[11] Karaoz E., Genc Z. S., Demircan P. C., Aksoy A., Duruksu G. (2010). Protection of rat pancreatic islet function and viability by coculture with rat bone marrow-derived mesenchymal stem cells. *Cell Death & Disease*.

[12] Duprez I. R., Johansson U., Nilsson B., Korsgren O., Magnusson P. U. (2011). Preparatory studies of composite mesenchymal stem cell islets for application in intraportal islet transplantation. *Upsala Journal of Medical Sciences*.

[13] Jung E. J., Kim S. C., Wee Y. M. (2011). Bone marrow-derived mesenchymal stromal cells support rat pancreatic islet survival and insulin secretory function in vitro. *Cytotherapy*.

[14] Rackham C. L., Dhadda P. K., Chagastelles P. C. (2013). Pre-culturing islets with mesenchymal stromal cells using a direct contact configuration is beneficial for transplantation outcome in diabetic mice. *Cytotherapy*.

[15] Montanari E., Meier R. P. H., Mahou R. (2017). Multipotent mesenchymal stromal cells enhance insulin secretion from human islets via N-cadherin interaction and prolong function of transplanted encapsulated islets in mice. *Stem Cell Research & Therapy*.

[16] Scuteri A., Donzelli E., Rodriguez-Menendez V. (2014). A double mechanism for the mesenchymal stem cells’ positive effect on pancreatic islets. *PLoS One*.

[17] de Souza B. M., Boucas A. P., dos Santos de Oliveira F. (2017). Effect of co-culture of mesenchymal stem/stromal cells with pancreatic islets on viability and function outcomes: a systematic review and meta-analysis. *Islets*.

[18] Arzouni A. A., Vargas-Seymour A., Rackham C. L. (2017). Mesenchymal stromal cells improve human islet function through released products and extracellular matrix. *Clinical Science*.

[19] Arzouni A. A., Vargas-Seymour A., Dhadda P. K. (2019). Characterization of the effects of mesenchymal stromal cells on mouse and human islet function. *Stem Cells Translational Medicine*.

[20] Ayenehdeh J. M., Niknam B., Rasouli S. (2017). Immunomodulatory and protective effects of adipose tissue-derived mesenchymal stem cells in an allograft islet composite transplantation for experimental autoimmune type 1 diabetes. *Immunology Letters*.

[21] Ren G., Rezaee M., Razavi M., Taysir A., Wang J., Thakor A. S. (2019). Adipose tissue-derived mesenchymal stem cells rescue the function of islets transplanted in sub-therapeutic numbers via their angiogenic properties. *Cell and Tissue Research*.

[22] Moher D., Liberati A., Tetzlaff J., Altman D. G., The PRISMA Group (2010). Preferred reporting items for systematic reviews and meta-analyses: the PRISMA statement. *International Journal of Surgery*.

[23] Balshem H., Helfand M., Schünemann H. J. (2011). GRADE guidelines: 3. Rating the quality of evidence. *Journal of Clinical Epidemiology*.

[24] Zlowodzki M., Poolman R. W., Kerkhoffs G. M., Tornetta P., Bhandari M., On behalf of the International Evidence-Based Orthopedic Surgery Working (2007). How to interpret a meta-analysis and judge its value as a guide for clinical practice. *Acta Orthopaedica*.

[25] Park K. S., Kim Y. S., Kim J. H. (2009). Influence of Human Allogenic Bone Marrow and Cord Blood–Derived Mesenchymal Stem Cell Secreting Trophic Factors on ATP (adenosine-5′-triphosphate)/ADP (adenosine-5′-diphosphate) Ratio and Insulin Secretory Function of Isolated Human Islets From Cadaveric Donor. *Transplantation Proceedings*.

[26] Park K. S., Kim Y. S., Kim J. H. (2010). Trophic molecules derived from human mesenchymal stem cells enhance survival, function, and angiogenesis of isolated islets after transplantation. *Transplantation*.

[27] Davis N. E., Beenken-Rothkopf L. N., Mirsoian A. (2012). Enhanced function of pancreatic islets co-encapsulated with ECM proteins and mesenchymal stromal cells in a silk hydrogel. *Biomaterials*.

[28] Jun Y., Kang A. R., Lee J. S. (2014). Microchip-based engineering of super-pancreatic islets supported by adipose- derived stem cells. *Biomaterials*.

[29] Karaoz E., Ayhan S., Okçu A. (2011). Bone marrow-derived mesenchymal stem cells co-cultured with pancreatic islets display *β* cell plasticity. *Journal of Tissue Engineering and Regenerative Medicine*.

[30] Kerby A., Jones E. S., Jones P. M., King A. J. (2013). Co-transplantation of islets with mesenchymal stem cells in microcapsules demonstrates graft outcome can be improved in an isolated-graft model of islet transplantation in mice. *Cytotherapy*.

[31] Kono T. M., Sims E. K., Moss D. R. (2014). Human adipose-derived stromal/stem cells protect against STZ-induced hyperglycemia: analysis of hASC-derived paracrine effectors. *Stem Cells*.

[32] Lin P., Chen L., Li D., Yang N., Sun Y., Xu Y. (2009). Dynamic analysis of bone marrow mesenchymal stem cells migrating to pancreatic islets using coculture microfluidic chips: an accelerated migrating rate and better survival of pancreatic islets were revealed. *Neuro Endocrinology Letters*.

[33] Lu Y., Jin X., Chen Y. (2010). Mesenchymal stem cells protect islets from hypoxia/reoxygenation-induced injury. *Cell Biochemistry and Function*.

[34] Rackham C. L., Dhadda P. K., Le Lay A. M., King A. J., Jones P. M. (2014). Preculturing islets with adipose-derived mesenchymal stromal cells is an effective strategy for improving transplantation efficiency at the clinically preferred intraportal site. *Cell Medicine*.

[35] Rackham C. L., Vargas A. E., Hawkes R. G. (2016). Annexin A1 is a key modulator of mesenchymal stromal cell-mediated improvements in islet function. *Diabetes*.

[36] Yamada S., Shimada M., Utsunomiya T. (2014). Trophic effect of adipose tissue–derived stem cells on porcine islet cells. *Journal of Surgical Research*.

[37] Yoshimatsu G., Sakata N., Tsuchiya H. (2013). Development of polyvinyl alcohol bioartificial pancreas with rat islets and mesenchymal stem cells. *Transplantation Proceedings*.

[38] Chen J., Ye Y., Liao L. (2013). Mesenchymal stem cells promote islet survival in vitro and function in vivo. *Cell R4*.

[39] Balamurugan A. N., Naziruddin B., Lockridge A. (2014). Islet product characteristics and factors related to successful human islet transplantation from the Collaborative Islet Transplant Registry (CITR) 1999-2010. *American Journal of Transplantation*.

[40] Balamurugan A. N., Loganathan G., Bellin M. D. (2012). A new enzyme mixture to increase the yield and transplant rate of autologous and allogeneic human islet products. *Transplantation*.

[41] Fraker C. A., Cechin S., Álvarez-Cubela S. (2013). A physiological pattern of oxygenation using perfluorocarbon-based culture devices maximizes pancreatic islet viability and enhances *β*-Cell function. *Cell Transplantation*.

[42] Rickels M. R., Liu C., Shlansky-Goldberg R. D. (2013). Improvement in *β*-cell secretory capacity after human islet transplantation according to the CIT07 protocol. *Diabetes*.

[43] Barton F. B., Rickels M. R., Alejandro R. (2012). Improvement in outcomes of clinical islet transplantation: 1999-2010. *Diabetes Care*.

[44] Carlsson P. O., Schwarcz E., Korsgren O., Le Blanc K. (2015). Preserved *β*-cell function in type 1 diabetes by mesenchymal stromal cells. *Diabetes*.

[45] Rackham C. L., Jones P. M. (2018). Potential of mesenchymal stromal cells for improving islet transplantation outcomes. *Current Opinion in Pharmacology*.

[46] Wang L., Qing L., Liu H. (2017). Mesenchymal stromal cells ameliorate oxidative stress-induced islet endothelium apoptosis and functional impairment via Wnt4-*β*-catenin signaling. *Stem Cell Research & Therapy*.

[47] D'Acquisto F., Perretti M., Flower R. J. (2008). Annexin-A1: a pivotal regulator of the innate and adaptive immune systems. *British Journal of Pharmacology*.

[48] Emamaullee J. A., Rajotte R. V., Liston P. (2005). XIAP overexpression in human islets prevents early posttransplant apoptosis and reduces the islet mass needed to treat diabetes. *Diabetes*.

[49] English K. (2016). Mesenchymal stem cells to promote islet transplant survival. *Current Opinion in Organ Transplantation*.

[50] Caplan A. I., Dennis J. E. (2006). Mesenchymal stem cells as trophic mediators. *Journal of Cellular Biochemistry*.

[51] Hu C., Li L. (2018). Preconditioning influences mesenchymal stem cell properties in vitro and in vivo. *Journal of Cellular and Molecular Medicine*.

[52] Kosaka N., Sudo N., Miyamoto A., Shimizu T. (2007). Vascular endothelial growth factor (VEGF) suppresses ovarian granulosa cell apoptosis in vitro. *Biochemical and Biophysical Research Communications*.

[53] Linnemann A. K., Blumer J., Marasco M. R. (2017). Interleukin 6 protects pancreatic *β* cells from apoptosis by stimulation of autophagy. *The FASEB Journal*.

[54] Marasco M. R., Conteh A. M., Reissaus C. A. (2018). Interleukin-6 reduces *β*-Cell oxidative stress by linking autophagy with the antioxidant response. *Diabetes*.

[55] Barshes N. R., Wyllie S., Goss J. A. (2005). Inflammation-mediated dysfunction and apoptosis in pancreatic islet transplantation: implications for intrahepatic grafts. *Journal of Leukocyte Biology*.

[56] Luo L., Badiavas E., Luo J. Z., Maizel A. (2007). Allogeneic bone marrow supports human islet *β* cell survival and function over six months. *Biochemical and Biophysical Research Communications*.

[57] Eich T., Eriksson O., Lundgren T. (2007). Visualization of early engraftment in clinical islet transplantation by positron-emission tomography. *The New England Journal of Medicine*.

[58] Duffy M. M., Ritter T., Ceredig R., Griffin M. D. (2011). Mesenchymal stem cell effects on T-cell effector pathways. *Stem Cell Research & Therapy*.

[59] Ben Nasr M., Vergani A., Avruch J. (2015). Co-transplantation of autologous MSCs delays islet allograft rejection and generates a local immunoprivileged site. *Acta Diabetologica*.

